# Serum S100B levels after meningioma surgery: A comparison of two laboratory assays

**DOI:** 10.1186/1472-6890-8-9

**Published:** 2008-09-19

**Authors:** Sharon Einav, Eyal Itshayek, Jeremy D Kark, Haim Ovadia, Carolyn F Weiniger, Yigal Shoshan

**Affiliations:** 1 The Intensive Care Unit of the Shaare Zedek Medical Center, affiliated with the Hebrew University, POB 3235, Jerusalem 91031, Israel; 2 The Department of Neurosurgery, Hadassah Hebrew University Medical Center, Jerusalem, Israel; 3 The Epidemiology Unit, Hadassah Hebrew University Medical Center, Jerusalem, Israel; 4 The Department of Neurology, Agnes Ginges Center for Human Neurogenetics, Hadassah Hebrew University Medical Center, Jerusalem, Israel; 5 The Department of Anesthesia, Hadassah Hebrew University Medical Center, Jerusalem, Israel; 6 The Department of Neurosurgery, Hadassah Hebrew University Medical Center, Jerusalem, Israel

## Abstract

**Background:**

S100B protein is a potential biomarker of central nervous system insult. This study quantitatively compared two methods for assessing serum concentration of S100B.

**Methods:**

A prospective, observational study performed in a single tertiary medical center. Included were fifty two consecutive adult patients undergoing surgery for meningioma that provided blood samples for determination of S100B concentrations. Eighty samples (40 pre-operative and 40 postoperative) were randomly selected for batch testing. Each sample was divided into two aliquots. These were analyzed by ELISA (Sangtec) and a commercial kit (Roche Elecsys^®^) for S100B concentrations. Statistical analysis included regression modelling and Bland-Altman analysis.

**Results:**

A parsimonious linear model best described the prediction of commercial kit values by those determined by ELISA (y = 0.045 + 0.277*x, x = ELISA value, R^2 ^= 0.732). ELISA measurements tended to be higher than commercial kit measurements. This discrepancy increased linearly with increasing S100B concentrations. At concentrations above 0.7 μg/L the paired measurements were consistently outside the limits of agreement in the Bland-Altman display. Similar to other studies that used alternative measurement methods, sex and age related differences in serum S100B levels were not detected using the Elecsys^® ^(p = 0.643 and 0.728 respectively).

**Conclusion:**

Although a generally linear relationship exists between serum S100B concentrations measured by ELISA and a commercially available kit, ELISA values tended to be higher than commercial kit measurements particularly at concentrations over 0.7 μg/L, which are suggestive of brain injury. International standardization of commercial kits is required before the predictive validity of S100B for brain damage can be effectively assessed in clinical practice.

## Background

Protein S100 is an acidic, disulfide-linked, dimeric, calcium-binding, low molecular weight protein. The beta subtype of this protein exists in astrocyte cells in relatively high concentrations. Rises in serum concentrations of S100B have been shown to relate to clinical evidence of CNS damage in the three accepted models of brain injury in humans-trauma [1-3], ischemia [4] and hypoxia [5]. Significantly higher concentrations of S100B have been demonstrated in brain death [3] and in non survivors from cardiopulmonary arrest, compared to survivors [5,6].

The value of using S100B as an indicator of neurological injury in the clinical setting is limited by the relatively high cost and lengthy performance time of the enzyme-linked immunosorbent assay (ELISA) (currently considered the best method of analysis), poor substantiation of reference levels, lack of standardization of serum S-100B testing and the paucity of data regarding the relationship between measurement methods. Most studies demonstrating the potential diagnostic value of serum S-100B in neurological diseases used an ELISA method (Sangtec Medical) [1,5,7,8]. Recently a kit for rapid quantification of S-100B in human serum has become available (Roche Pharmaceuticals, Elecsys^®^).

The current study was designed to quantitatively compare the commercially available Elecsys^® ^assay for serum S100B protein concentrations with the gold standard ELISA method and to examine whether this test detects sex- and age-related differences in serum S100B protein concentrations similar to those demonstrated with ELISA by Gazzolo et al. [9] and Nyberg et al. [10] in pediatric populations.

## Methods

### Sampling technique

Following institutional review board approval (Hadassah Hebrew University Medical Center, reference number 14–19/12/03) as well as individual patient informed consent, 52 consecutive adult patients aged 18–80 who underwent supratentorial meningioma surgery over a 10 month period provided pre- and post operative blood samples for analysis of S100B concentrations.

Blood samples were drawn into sterile, preservative-free vacuum containers for the evaluation of S100B in the perioperative period as described elsewhere [11]. For the purpose of the current study, samples drawn prior to surgery (after insertion of an intravenous cannula upon admission) and immediately after surgery were used. These sampling times were selected based on the assumption that the broadest range of serum concentrations would be obtained between the baseline measurement and the measurement at maximal proximity to surgical insult to the central nervous system. Previous data have demonstrated that at this time S100B concentrations are highly correlated with post-craniotomy neurological deterioration and unfavorable 6-month outcome [12]. Since the ELISA array allows batch testing of forty samples at a time, eighty samples were selected at random for the current study, 40 pre-operative and 40 postoperative.

### Laboratory testing and workup of blood samples

Blood samples were first allowed to clot for 30 min at room temperature and then centrifuged. Following centrifugation for 15 minutes at 2000 rpm, serum samples were stored at -70°c for up to ~3 months for batch analysis. The serum samples were then thawed to room temperature, divided into two aliquots and analyzed in parallel by the ELISA and Elecsys^® ^methods. Diluted serum was used for higher values in both assays.

#### Assessment by ELISA

Testing was performed using the Sangtec 100 ELISA immunosorbent assay for quantitative measurement of S100B protein in human serum (DiaSorin Inc., Stillwater, Minnesota, USA). Each sample was incubated together with an appropriate marker. Washout was performed with a buffer and tetramethylbenzidine was added as a substrate. Following further incubation a tetramethylbenzidine-arresting substance was added. Spectrophotometric reading of light absorbance at 450 nm was performed. Calculation of the result was performed using a cubic spline algorithm. The calculation range is accurate to a measured concentration of 5 μg//L.

#### Assessment by Elecsys^®^

Testing was performed using the Roche Elecsys^® ^S100 reagent kit (assay duration 18 minutes, measuring range 0.005–39 μg/L, cross reactivity against S100α < 1%). Less than 24 hr prior to testing calibration was performed per reagent kit and control values were determined to be within the limits required for calibration (0.206 μg/L and 2.54 μg/L). The test kit is based on the sandwich principle; the 1^st ^incubation is performed with a biotinylated monoclonal S100-specific antibody and a monoclonal S100-specific antibody labeled with a ruthenium complex react to form a sandwich complex. The 2^nd ^incubation: After addition of streptavidin-coated microparticles the complex becomes bound to the solid phase via interaction of biotin and streptavidin. The reaction mixture is aspirated into the measuring cell where the microparticles are magnetically captured onto the surface of the electrode. Unbound substances are then removed with ProCell. Application of a voltage to the electrode then induces chemiluminescent emission which is measured by a photomultiplier. Results are determined via a calibration curve which is instrument-specifically generated by 2-point calibration and a master curve provided via the reagent barcode.

The two laboratory analyses were performed by different technicians in different times. Both were blinded to the results of the alternative method.

### Statistical analysis

In the first stage, several regression models were used to examine the Elecsys^® ^method as a function of the ELISA method using all the samples. These included linear, quadratic, cubic, logarithmic, inverse, compound, power, s and growth. Among those models with the best fit (judged by the R^2 ^value) the most parsimonious was chosen. In the second stage, Bland-Altman analyses [13] were used to compare the quantification of S100 between Elecsys^® ^and ELISA and to detect the existence of systematic errors. The statistical analyses were performed using SPSS 12 software (SPSS Inc, Chicago, IL). The study endpoint was examination of the relationship between the two test methods.

Preoperative samples were used for detection of age- and sex-related changes in serum S-100B levels using the Elecsys^® ^assay. Statistical analyses included the Mann-Whitney test for comparison between sexes, the Spearman correlation coefficient to evaluate correlation between serum S100B and age and post-hoc ANOVA to test for differences between serum S100B levels and age groups.

## Results

### Quantitative comparison between the two methods

**Figure 1:**
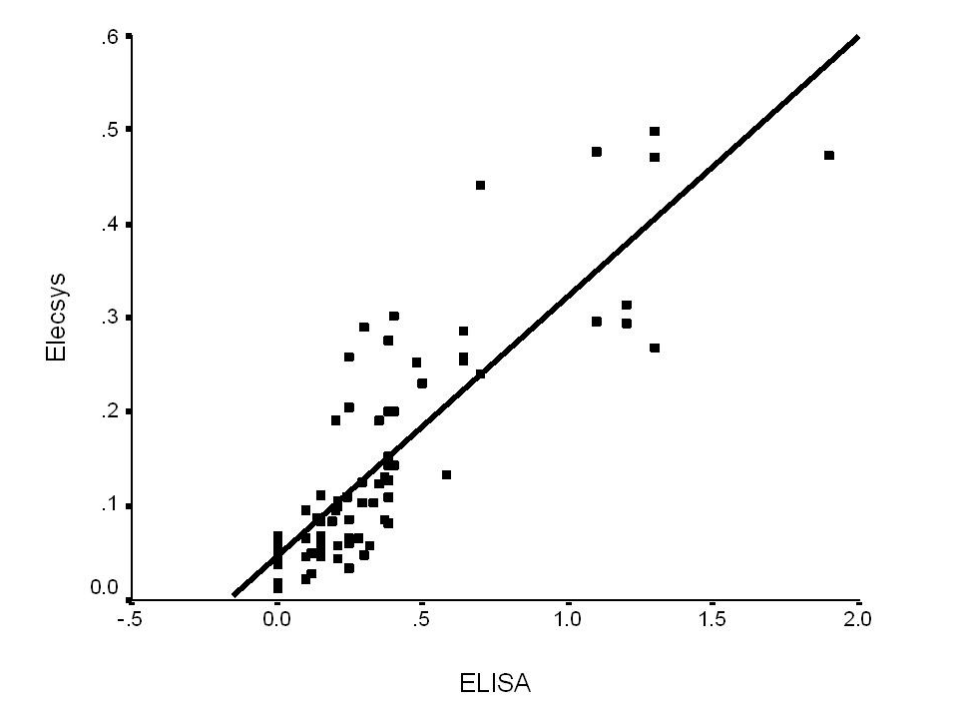
**S100B measured using ELISA and Elecsys^®^, with regression line**. The formula for the regression line of the relationship between the two test methods was y = 0.045269 + 0.276976*x, with x = ELISA value. All serum concentrations are given in μg/L.

The best fitting models that assessed Elecsys^® ^values as a function of ELISA values were linear, quadratic and cubic regressions, with R^2^values of 0.732, 0.752 and 0.753 respectively. As the R^2 ^values were very similar, the more parsimonious (in terms of the number of parameters in the model) linear model was selected to represent the relationship between the two test methods. The formula for the regression line of the relationship between the two test methods was y = 0.045269 + 0.276976*x, with x = ELISA value (Figure 1).

**Figure 2:**
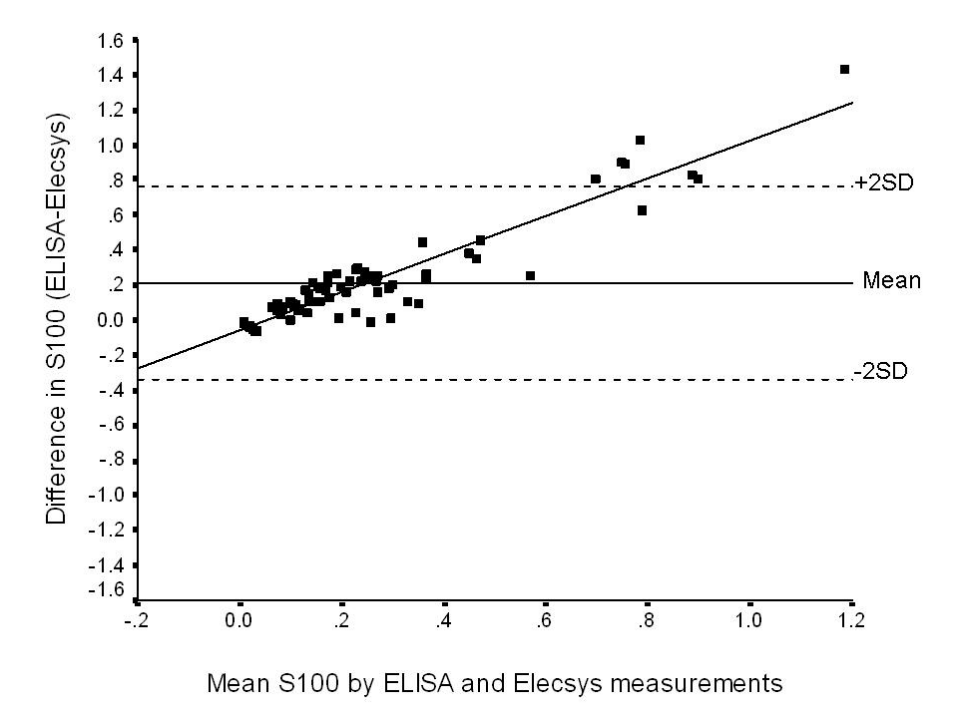
**Bland and Altman comparison of serum S100 concentrations as measured by ELISA and Elecsys^® ^measurements**. The x-axis represents the average measurement and the y-axis represents the difference (in μg/L) in measurements when these were made using ELISA and Elecsys^® ^methods. The mean difference ± 2 SDs are represented by horizontal complete and dotted lines respectively. The value of the R^2 ^for the regression line is 0.88.

The mean difference in measurement between the two tests (ELISA minus Elecsys^®^) was 0.214 ± 0.277 μg/L. However, this value is determined by the distribution of the measured concentrations (i.e. the patient mix) with the discrepancy being a linear function of the S100B concentration. The Bland-Altman analysis clearly demonstrated that the values determined by ELISA were higher than by Elecsys^®^, with the discrepancy increasing in linear mode as the S100B concentration increased so that the ELISA values markedly exceeded the Elecsys^® ^values at the higher concentrations (Figure 2). The degree of discrepancy was very large (>2 SD) at values above 0.7 μg/L (i.e. in the region of values suggestive of brain injury).

### Relationship between S100B and age

**Figure 3:**
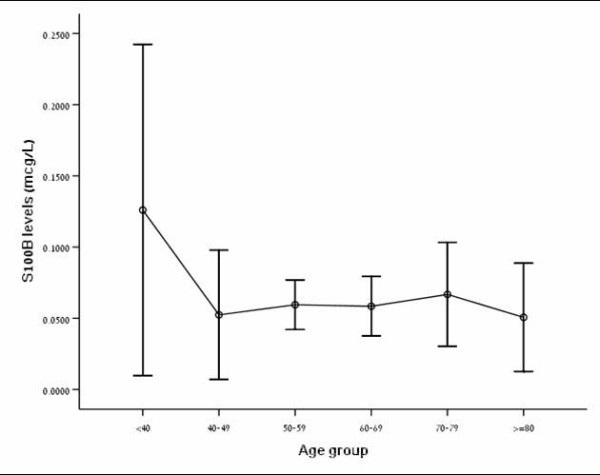
**Relationship of S100B levels with age**. Error bar for preoperative serum S100B levels (μg/L) by decade for patients with meningioma (n = 40). Data are presented together for male and female patients since no sex differences were found.

No statistically significant sex difference was noted in serum S100B concentrations measured by the Elecsys^® ^method (p = 0.643), nor was there a correlation between S100B concentration and age (p = 0.728) (figure 3).

## Discussion

The current study provides a quantitative comparison of two tests for measuring the serum concentration of S100B; ELISA and the commercially available Elecsys^®^. In terms of R^2 ^and the number of parameters in the model, the linear model provided the best fit for the relationship between the results achieved by these tests. However, in our hands ELISA measurements were higher than Elecsys^® ^measurements and particularly so at the higher values, where the differences in the measurements made using the two methods were consistently outside the limits of agreement (2 SDs) as seen in the Bland-Altman display.

Mussack et al. [14] performed a similar analysis in a group of patients undergoing carotid revascularization. With the exception of two patients with ongoing damage, the results of ELISA testing in their series were curtailed at 0.4 μg/L whereas the current paper describes ranges up to 1.2 μg/L. In contrast with our study, Mussack et al. found small, inconsistent mean differences between the methods and little or no change in the difference between methods with increasing S100 concentrations. It is precisely the divergence at higher values reported in our paper (i.e. those more likely to be associated with brain damage) that may cause misclassification of patient status and prognosis; hence the need for standardization. The current paper relates to brain damage as a result of an intracranial operative procedure and may therefore be more pertinent to the range of test values to be expected in direct injury to the brain.

Sex and age related differences in serum S100B levels were not detected using the Elecsys^®^. This result is consistent with other studies that used alternative measurement methods which demonstrated no sex and age differences in normal adult populations [10,15,16], contrary to pediatric populations [9,10].

There are several subunits of S100 proteins [17], many of which are expressed selectively by specific cell types. The B subunit of the S100 protein (S100B) is found in particularly high concentrations in astroglial cells of the central nervous system and is therefore often referred to as Astrocyte Derived Growth Factor [18,19]. S100B is implicated in the coordinated development and maintenance of the central nervous system; it stimulates differentiation of immature neurons [18-23], promotes cell survival [23,24], induces neurite extension [25] and enhances glial cell proliferation. Observations of higher S100B concentrations humans in infants and adolescence have led some researchers to suggest that the release of this neurotrophic factor decreases as the brain matures [9,10].

Serum S100B concentrations rise in clinical situations representing the three models of human brain injury – trauma [1-3], ischemia [4,26] and hypoxia [5,27]. The degree of elevation is probably related to the severity of blood-brain barrier disruption [28,29]. Although S100B is eliminated by the kidneys, in-vivo studies have not shown a detectable rise in S100B concentration in renal failure, probably due to its estimated 2 hour biological half life [30]. Prolonged post-insult elevation of serum S100B concentrations therefore most probably reflects ongoing central nervous system damage.

Routine use of S100B for early diagnosis of brain injury remains limited for a number of reasons. Lack of specificity for the biologically active dimeric form of the S-100B molecule using the actually available assays is a major problem. This protein can be released from extracerebral tissue, particularly after surgery [31], potentially confounding the association with brain injury and resulting in misclassification. S100B can be released from the heart or mediastinum [32,33] and exists in adipose tissue [34,35], skin, testes [34], skeletal muscle [36] and placental tissue [37,38], albeit in significantly lower concentrations than in brain tissue. The definition of "normal" values in humans has yet to be established in each assay and cutoff values for diagnosis and quantification of the presence and extent of brain injury remain undefined. Clinical applicability is also limited by the lack of standardization. Different laboratories using the same kit produced substantially different values [5,6,39,40] and between-kit variance may be even larger.

Elecsys^® ^analysis requires an electrochemiluminescence measuring cell. Apart from the initial centrifuging, testing is fully automated; thus only a few minutes of laboratory staff time are needed for analysis. The reagent kit suffices for up to 100 tests. Calibration is required once monthly and requires two calibration standards in duplicate (4 tests). Two control tests are required daily (2 tests). The real cost of any test is subject to kit yield which depends on several variables (e.g. daily number of tests, calibration and quality control procedures). Calculated yield may be therefore be greatly affected by internal laboratory regulations. Kit yield is close to maximal at 20 tests per day, at which point additional costs are almost negligible (Table 1). In 2008 the local price for an Elecsys S100 kit was 1528€. Thus, the theoretical price per test was 15.28€. For a daily routine of 5 reported results (kit yield 0.6451) the real price per test was 23.69€ and for a daily routine of 12 reported results (kit yield 0.8235) the real price per test was 18.55€. Additional costs of calibrator and control materials should be noted: The estimated consumption of S100 calibrator (price 80.00€/pack) and control (price 189.00€/pack) is 2 and 3 packs/year accordingly, yielding additional estimated costs of 727€/year. Analysis of S100B levels by ELISA requires specialized equipment, a substantial amount of laboratory technician time (approximately 90 minutes per test) and the test kit (which costs 1257€) usually allows for performance of 40 tests. ELISA measurement remains the gold standard for S100B measurements. However, widespread use of ELISA remains limited by cost/benefit considerations, mainly due to its time-consuming nature.

**Table 1:** Elecsys^® ^rack-pack yield for 28 days.

**n tests**					
	
**For clinical use daily**	**For clinical use monthly**	**For calibration**	**For Quality Control**	**Total**	**Kit yield**
1	28	16	56	101	0.28
2	56	16	56	130	0.43
3	84	16	56	159	0.53
4	112	16	56	188	0.60
5	140	16	56	217	0.65
10	280	16	56	362	0.77
15	420	4	56	495	0.85
20	560	4	56	640	0.88
25	700	4	56	785	0.89
30	840	4	56	930	0.90
40	1120	4	56	1220	0.92
50	1400	4	56	1510	0.93
60	1680	4	56	1800	0.93
70	1960	4	56	2090	0.94
80	2240	4	56	2380	0.94
90	2520	4	56	2670	0.94
100	2800	4	56	2960	0.95

Data are correct provided manufacturer specifications and recommendations are followed. Kit yield is close to maximal at 20 tests per day, at which point additional costs are close to negligible.

This study provides an assessment of the relationship between two currently used laboratory methods but does not address the diagnostic accuracy of either (i.e. sensitivity or specificity, optimal cutoff points). The inequality demonstrated between the tests at higher S100 values may have important implications for its use both as a prognostic marker and for clinical diagnosis as different cutoff points may be needed for the different test kits – an overtly undesirable clinical situation. Coefficients of variation are not given for the measurement methods. One should also exercise caution in extrapolating the findings of this study to concentrations of S100B higher than those included in the study.

Clinical tools for early diagnosis of brain damage in critically ill patients are lacking. Physical examination and intuitive prediction are often the mainstays for both diagnostic and treatment decisions in these patients. This is a far from optimal situation in the intensive care environment where judicious allocation of hospital resources is required; survival with overwhelming residual neurological disability and absent or poor quality of life following insult to the central nervous system incurs expensive in- and out-of-hospital therapy and high indirect costs [40-45].

Despite its limitations, serum S100B, one of the most studied biomarkers of brain damage in the clinical setting, is currently the only one of which we are aware that holds promise as an early marker of brain damage and a predictor of probability of survival and severe neurological disability. Our study points to some shortcomings of currently available measurement methods and to the need for better definition of reference values and for international standardization of commercial kits so that the predictive validity of S100B can be effectively assessed in clinical practice.

## Conclusion

Although a general linear relationship exists between serum S100B concentrations measured by ELISA and a commercially available kit, ELISA measurements tended to be higher than commercial kit measurements particularly in concentrations over 0.7 μg/L, which are suggestive of brain injury. International standardization between methods of S100B measurements is required to enable effective assessment of the predictive validity of S100B in clinical practice.

## Competing interests

The authors declare that they have no competing interests.

## Authors' contributions

SE, YS and JDK are responsible for conceiving and designing the study. EI acquired the data. SE performed data analysis under expert statistical supervision and drafted the paper. JDK assisted in interpretation of the data and provided critical revision of the article together with HO and CFW.

## Pre-publication history

The pre-publication history for this paper can be accessed here:


